# Transcriptomic and proteomic pathways of diabetic and non-diabetic mitochondrial transplantation

**DOI:** 10.1038/s41598-022-25858-z

**Published:** 2022-12-21

**Authors:** Ilias P. Doulamis, Rio S. Nomoto, Aspasia Tzani, Xuechong Hong, Thomas Duignan, Aybuke Celik, Pedro J. del Nido, James D. McCully

**Affiliations:** 1 Department of Cardiac Surgery, Boston Children’s Hospital, 300 Longwood Avenue Enders-361.2, Boston, MA 02115 USA; 2 Brigham and Women’s Hospital Heart and Vascular Center, Boston, MA USA; 3 Harvard Medical School, Boston, MA USA

**Keywords:** Cardiology, Medical research

## Abstract

Reduced mitochondrial function increases myocardial susceptibility to ischemia–reperfusion injury (IRI) in diabetic hearts. Mitochondrial transplantation (MT) ameliorates IRI, however, the cardioprotective effects of MT may be limited using diabetic mitochondria. Zucker Diabetic Fatty (ZDF) rats were subjected to temporary myocardial RI and then received either vehicle alone or vehicle containing mitochondria isolated from either diabetic ZDF or non-diabetic Zucker lean (ZL) rats. The ZDF rats were allowed to recover for 2 h or 28 days. MT using either ZDF- or ZL-mitochondria provided sustained reduction in infarct size and was associated with overlapping upregulation of pathways associated with muscle contraction, development, organization, and anti-apoptosis. MT using either ZDF- or ZL-mitochondria also significantly preserved myocardial function, however, ZL- mitochondria provided a more robust long-term preservation of myocardial function through the mitochondria dependent upregulation of pathways for cardiac and muscle metabolism and development. MT using either diabetic or non-diabetic mitochondria decreased infarct size and preserved functional recovery, however, the cardioprotection afforded by MT was attenuated in hearts receiving diabetic compared to non-diabetic MT.

Type 2 diabetes (T2D) affects 1 out of 10 people worldwide and is associated with a fourfold higher risk for mortality following myocardial ischemia reperfusion injury (IRI). The increased susceptibility of the diabetic myocardium to IRI has been shown to be directly related to mitochondrial dysfunction. Our studies and those by others have demonstrated that in the diabetic heart, mitochondrial respiratory capacity and ATP production are greatly reduced as compared to mitochondria from non-diabetic hearts^1–5^.

In previous reports, we have demonstrated that autologous mitochondrial transplantation, in which viable respiration competent mitochondria, isolated from non-ischemic tissue from the patient’s own body, provides a methodology to augment or enhance mitochondria damaged from IRI. The therapeutic safety and efficacy of mitochondrial transplantation has been demonstrated in a series of in vitro and in vivo animal studies^3–12^ and in a phase I clinical trial^13,14^. These studies have demonstrated that mitochondrial transplantation significantly enhances post-ischemic myocardial function and significantly reduces post-ischemic myocardial infarct size.

In a recent report we examined the efficacy of mitochondrial transplantation in an ex vivo isolated perfused Zucker diabetic fatty (ZDF) rat heart model^2^. In these experiments we sought to determine the cardioprotective efficacy of mitochondrial transplantation using diabetic mitochondria with reduced respiratory capacity and ATP production, as compared to viable non-diabetic mitochondria. These preliminary studies showed that both diabetic and non-diabetic mitochondrial transplantation provided equivalent post-ischemic infarct size reduction and functional recovery at 2 h recovery; however, we did note that total tissue ATP content was significantly increased in diabetic hearts that received non-diabetic mitochondria^2^. This observed difference led us to speculate that long-term efficacy may be compromised when using mitochondria isolated from diabetic tissue.

In this report, we extend our previous investigations using an in situ Zucker diabetic fatty (ZDF) rat model of IRI to explore the efficacy of mitochondrial transplantation, comparing the cardioprotection offered by diabetic and non-diabetic mitochondria. We also investigate the mechanistic pathways involved in these models using multiplex, RNA-Seq and SOMAscan analyses (Fig. 1).

**Figure 1:**
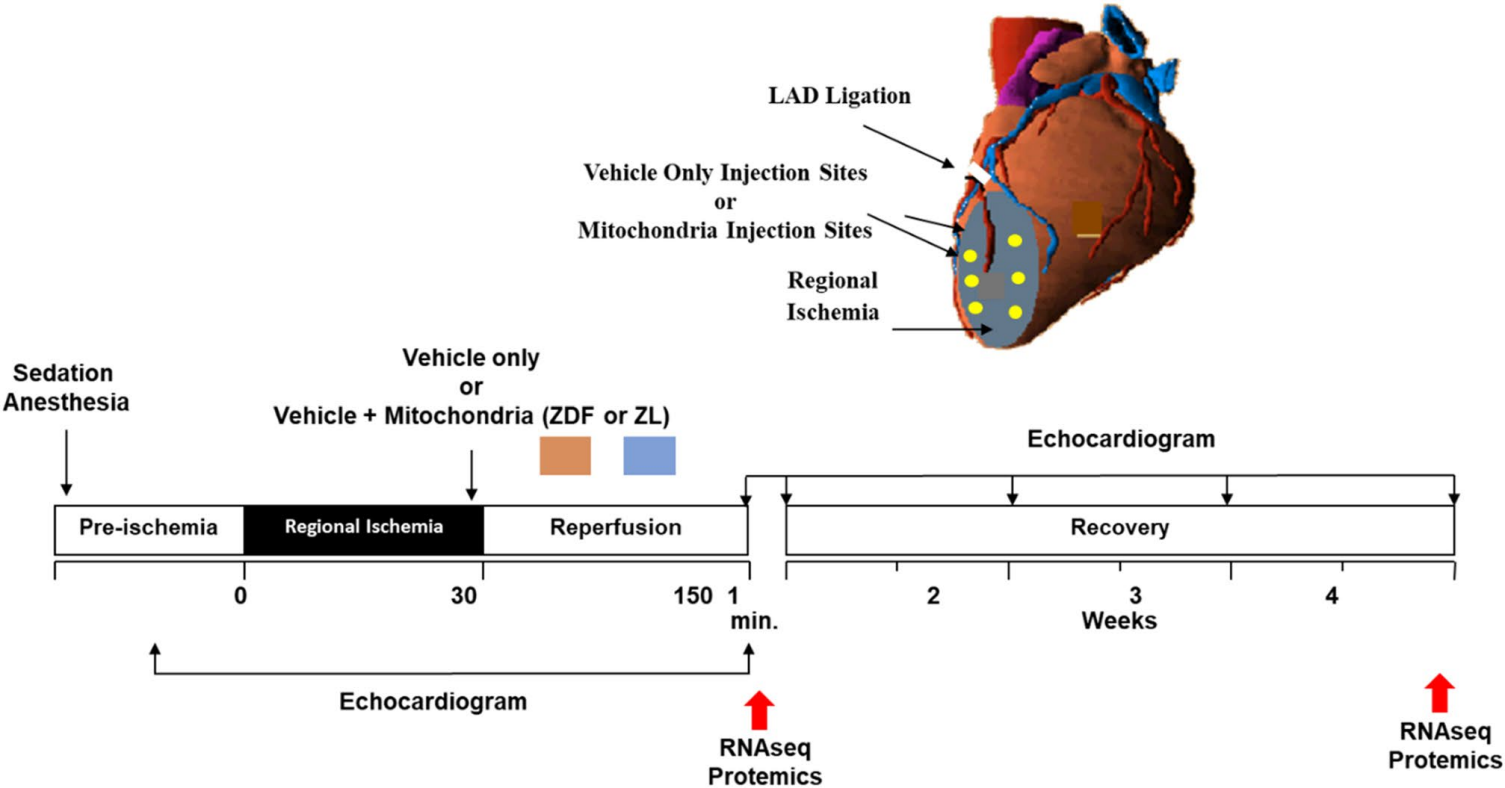
Experimental Model: ZDF rats (male, 14 weeks) were sedated and intubated. A left lateral thoracotomy was performed, and the heart was exposed. Regional ischemia was achieved by temporarily snaring the LAD. Following 30 min of ischemia, the snare was released, and 6 × 50 uL injections of vehicle solution alone (Vehicle) or vehicle solution containing mitochondria (1 × 10^6^) isolated from either diabetic ZDF rat pectoralis major (diabetic ZDF-mitochondria) or mitochondria (1 × 10^6^) isolated from non-diabetic Zucker lean rats (non-diabetic ZL-mitochondria) were delivered by epicardial injection into six pre-selected sites in the area-at risk, using a 1 mL tuberculin syringe with a 32-G needle. The ZDF rats were allowed to recover for 2 h or 28 days.

## Results

### ZDF rats maintained their diabetic phenotype throughout the study

**Figure 2:**
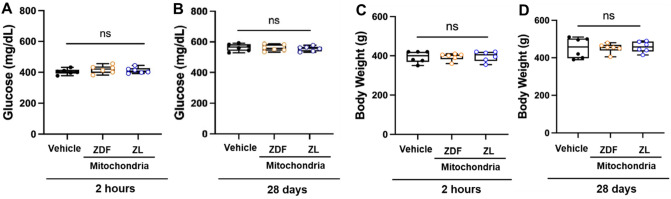
ZDF rat diabetic phenotype. Blood glucose levels (mg/dL) (**A**,**B**) and body weight (g) (**C**,**D**) for ZDF rats receiving vehicle alone (Vehicle), or diabetic ZDF- or non-diabetic ZL-mitochondria at 2 h (**A**,**C**) and at 28 days recovery (**B**,**D**). No significant differences in blood glucose, body weight or heart weight was observed within or between groups at 2 h or at 28 days recovery. Blood glucose and body weight were significantly increased at 28 days as compared to 2 h recovery. These data suggest that the observed effects of Vehicle, diabetic ZDL-and non-diabetic ZL-mitochondria transplantation are glucose independent.

Blood glucose levels (mg/dL), body weight (g) and heart weight (g) were measured to confirm the presence and the progression of T2D (Fig. 2). Blood glucose levels (mg/dL) were 404 ± 17.7 mg/dl, 412 ± 19.2 mg/dl and 419 ± 25.9 mg/dl at 2 h recovery and 564 ± 9.6 mg/dl, 555 ± 6.9 mg/dl and 56 ± 8.6 mg/dl at 28 days recovery (Fig. 2A,B) for ZDF rats in Vehicle and ZDF- and ZL-mitochondria groups, respectively. No significant difference in blood glucose was observed between groups at 2 h or 28 days recovery (*P* = 0.496 and *P* = 0.790, respectively), however, blood glucose was significantly increased in all groups (*P* < 0.001) at 28 days recovery as compared to 2 h recovery.

Body weight was 394 ± 12.3 g, 395 ± 7.5 g and 398 ± 10.7 g at 2 h recovery, for Vehicle, ZDF- and ZL-mitochondria groups respectively (Fig. 2C) and was 453 ± 22.4 g, 452 ± 10.3 g and 458 ± 11.9 g at 28 days recovery, respectively (Fig. 2D). No significant difference in body weight was observed between groups at 2 h or 28 days recovery (*P* = 0.972 and *P* = 0.961, respectively), however, body weight was significantly increased (*P* < 0.001) at 28 days as compared to 2 h recovery.

Heart weight at 28 days recovery was 1.58 ± 0.04 g, 1.53 ± 0.08 g and 1.55 ± 0.11 g for Vehicle, ZDF- and ZL-mitochondria groups, respectively. There was no significant difference in heart weight observed between groups (*P* = 0.872). The diabetic phenotype of the rats was maintained throughout the experimental period as shown by elevated body weight and whole blood glucose at 28 days recovery. All rats completed the experimental protocol. There were no animal losses in any group.

### Transplanted mitochondria were evident within the cardiomyocytes at 28 days and increased ATP content

**Figure 3:**
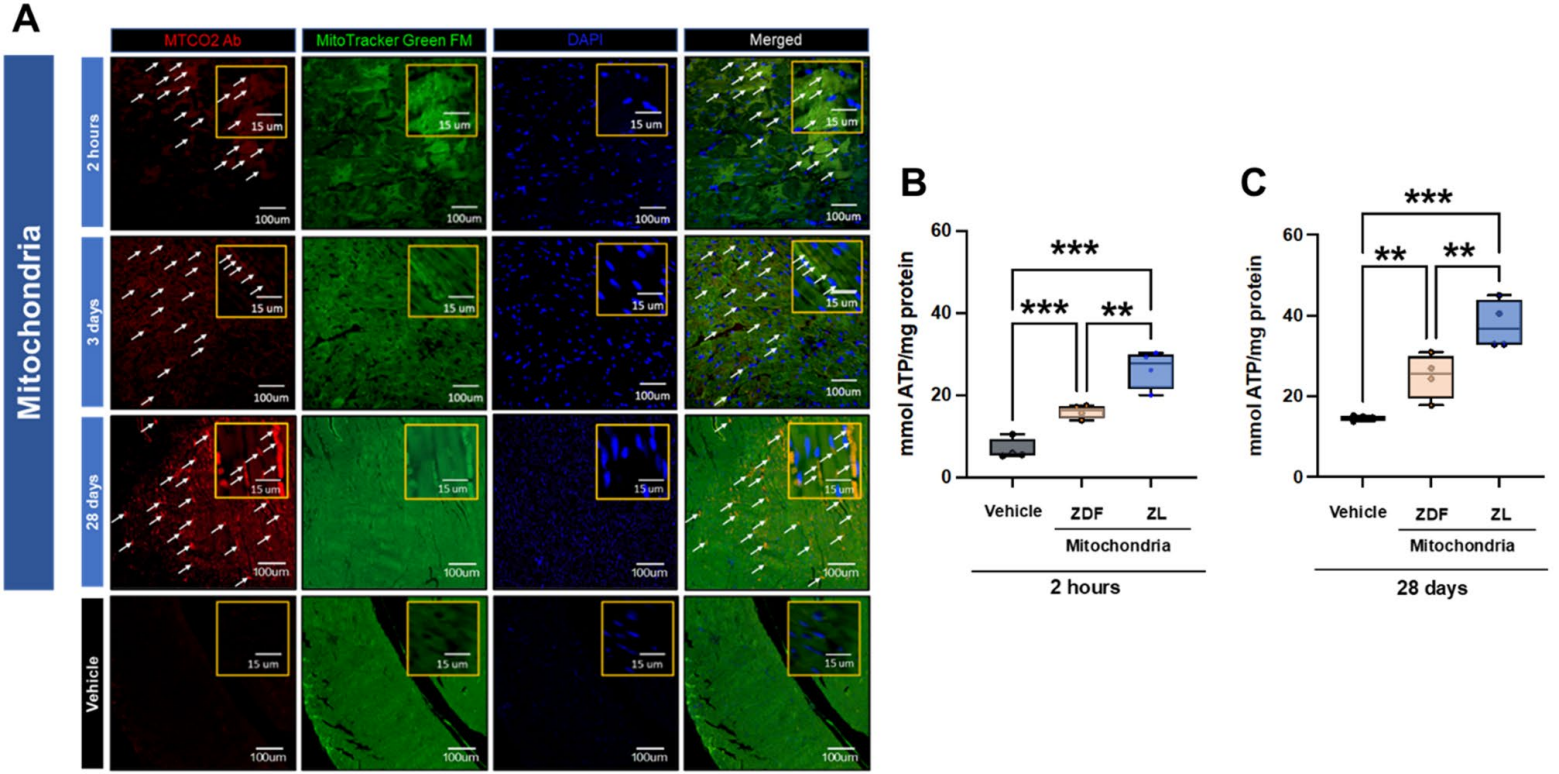
Localization, uptake, and function of transplanted mitochondria. Representative immunofluorescent images (**A**) from ZDF rat hearts following direct injection of vehicle alone (Vehicle) or vehicle containing human mitochondria (Mitochondria) to the hearts of ZDF rats following 30 min of regional ischemia and 2 h, 3 days and 28 days recovery. Human mitochondria were used only to confirm mitochondrial uptake. The use of human mitochondria in a rodent model allows for the differentiation between native rodent mitochondria and transplanted human mitochondria based on immune reactivity to a monoclonal anti-human mitochondria antibody (MTC02). Human mitochondria are shown labelled with human mitochondrial MTCO2 antibody (MTCO2ab, red). Mito-Tracker Green FM was used to show the injected mitochondria and in situ mitochondria. Nuclei were stained using DAPI (blue). Merged images are shown on the far right. Images are shown enlarged and with specific areas for each image enlarged to show detail. Enlarged images are boxed in gold. Transplanted human mitochondria are indicated by arrows. Mitochondria were observed within and around cardiomyocytes. Scale bars are 15 um and 100 um in length. Total tissue ATP content in the area-at-risk mmol/mg protein in vehicle hearts and in hearts receiving diabetic ZDF or non-diabetic ZL-mitochondria at 2 h (**B**) and 28 days (**C**) recovery. All results are shown as the mean plus and minus the standard error of the mean for n = 6 for each group. Statistical differences are shown as *p < 0.05, **p < 0.01, ***p < 0.001.

Our results show the transplanted mitochondria are present in the heart within and between cardiomyocytes within the area-at-risk at 2 h, 3 days and 28 days recovery. (Fig. 3A).

Total tissue ATP content in the area-at-risk was 6.8 ± 1.2 mmol/mg protein in Vehicle hearts at 2 h recovery. In contrast, in hearts receiving diabetic ZDF- or non-diabetic ZL-mitochondria, total tissue ATP content in the area-at-risk was significantly increased to 16.0 ± 0.8 and 26.4 ± 2.3 mmol/mg protein/mg tissue, respectively (*P* < 0.001 each vs. Vehicle). Total tissue ATP content in the area-at-risk was significantly increased in hearts receiving non-diabetic ZL- as compared to hearts receiving diabetic ZDF-mitochondria (*P* < 0.01) (Fig. 3B). At 28 days recovery, total tissue ATP content in the area-at-risk in vehicle hearts was 14.6 ± 0.3 mmol/mg protein/mg tissue. Total tissue ATP content in the area-at-risk was significantly increased (*P* < 0.01 vs Vehicle) to 24.9 ± 5.5 mmol/mg protein/mg tissue in hearts receiving diabetic ZDF-mitochondria (Fig. 3C).

In hearts receiving non-diabetic ZL-mitochondria, total tissue ATP content in the area-at-risk was significantly increased (*P* < 0.01 vs Vehicle and ZDF-mitochondria) to 37.8 ± 6.1 mmol/mg protein/mg tissue (Fig. 3B,C).

### Non-diabetic ZL- is superior to diabetic ZDF-mitochondrial transplantation for long-term functional recovery

**Figure 4:**
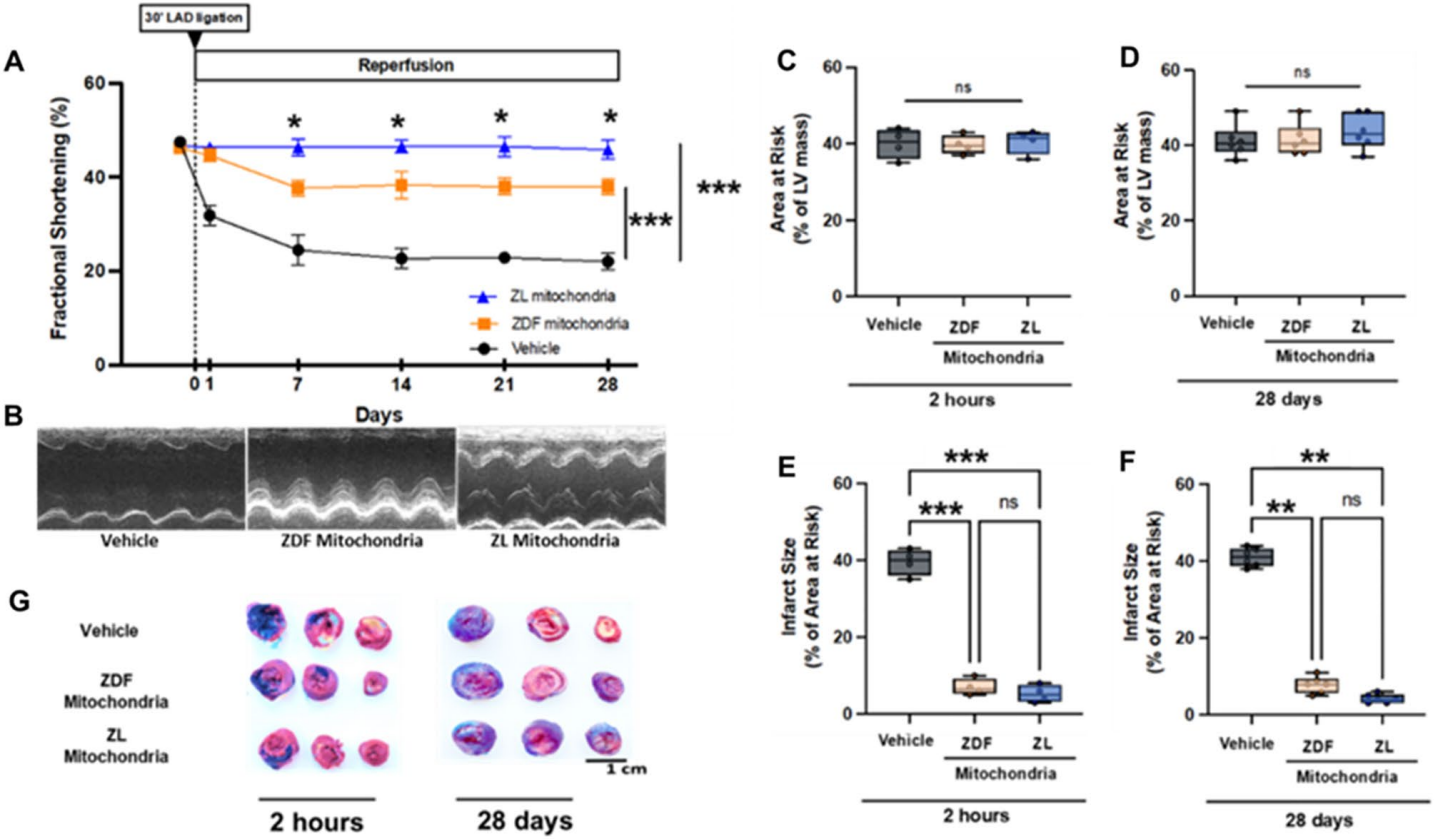
Myocardial function and viability. Left ventricular fractional shortening (**A**), determined by echocardiography in Vehicle hearts and in hearts receiving diabetic ZDF- or non-diabetic ZL-mitochondria at day 0 (baseline) and at 1, 7,14, 21 and 28 days recovery. Representative M mode at 28 days recovery is provided **(B)**. Area at risk as percentage of left ventricular mass in Vehicle hearts and in hearts receiving diabetic ZDF- or non-diabetic ZL -mitochondria following 30 min regional ischemia at 2 h (**C**) and 28 days recovery (**D**). Infarct size as % of area at risk in Vehicle hearts and in hearts receiving diabetic ZDF- or non-diabetic ZL -mitochondria following 30 min regional ischemia at 2 h (**E**) and 28 days recovery (**F**). Representative images of ZDF rat hearts stained with 1% triphenyltetrazolium chloride (TTC) for hearts receiving vehicle alone, diabetic ZDF- or non-diabetic ZL -mitochondria, following 30 min regional ischemia and at 2 h and 28 days recovery (**G**). White areas show myocardial necrosis. Brick red areas show viable tissue. Scale in cm is shown. All results are shown as the mean plus and minus the standard error of the mean for n = 6 for each group. Statistical differences are shown as * p < 0.05, ** p < 0.01, ***p < 0.001; ns: not significant.

Left ventricular fractional shortening was determined by echocardiography. Fractional shortening at day 0 was 47.5 ± 1.0%, 46.2 ± 1.2% and 46.7 ± 1.0% for Vehicle, ZDF- and ZL-mitochondria groups respectively. There was no difference in fractional shortening within or between groups at day 0 (Fig. 4A). Following 30 min. regional ischemia and 1 day recovery fractional shortening was significantly decreased (*P* < 0.001) to 31.8 ± 2.1% in Vehicle hearts as compared to pre-ischemia and to hearts receiving either diabetic ZDF- (44.7 ± 1.4%), or non-diabetic ZL-mitochondria (46.3 ± 0.5%). There was no significant difference in fractional shortening observed between hearts receiving either diabetic ZDF- or non-diabetic ZL-mitochondria as compared to day 0 (*P* = 0.999).

At 7, 14, 21 and 28 days recovery, fractional shortening in Vehicle hearts was significantly decreased (*P* < 0.001) as compared to day 0 and to hearts receiving either diabetic ZDF- or non-diabetic ZL-mitochondria (Fig. 4A).

Fractional shortening at 7, 14, 21, and 28 days recovery was significantly decreased (P < 0.001 for each) as compared to day 0 in hearts receiving diabetic ZDF-mitochondria but remained significantly increased (*P* < 0.001) as compared to vehicle hearts (Fig. 4A).

In hearts receiving non-diabetic ZL-mitochondria, fractional shortening at 7, 14, 21 and 28 days recovery was not significantly different from day 0 (*P* < 0.001) and was significantly increased (*P* < 0.05 for each) as compared to hearts receiving diabetic ZDF-mitochondria at 7, 14, 21, and 28 days recovery (Fig. 4A,B).

Fractional shortening at 28 days recovery was 22.0 ± 1.8% in Vehicle hearts, 38.0 ± 1.7% hearts receiving diabetic ZDF-mitochondria and 45.8 ± 2.0% in hearts receiving non-diabetic ZL-mitochondria (Vehicle vs ZDF-mitochondria = *P* < 0.001; Vehicle vs ZL-mitochondria = *P* < 0.001, ZDF-mitochondria vs Z-mitochondria = *P* < 0.001).

### Diabetic ZDF- and non-diabetic ZL-mitochondrial transplantation are equivalent in reducing myocardial infarct size

Myocardial infarct size was expressed as a percentage of the area-at-risk for each heart. The area-at-risk was 40.0 ± 3.9, 39.8 ± 2.5 and 40.5 ± 3.1% LV mass at 2 h recovery, and 42.3 ± 4.4, 41.5 ± 4.1, and 43.7 ± 4.7, % LV mass at 28 days recovery for hearts receiving Vehicle, diabetic ZDF- and non-diabetic ZL-mitochondria, respectively. There was no significant difference in the area-at-risk within or between groups for hearts receiving Vehicle, diabetic ZDF- or non-diabetic ZL-mitochondria at 2 h and at 28 days recovery (*P* = 0.999) (Fig. 4C,D).

Myocardial infarct size following 30 min. regional ischemia and 2 h recovery was 33.6 ± 1.7% LV area-at-risk in Vehicle hearts. In contrast, in hearts receiving diabetic ZDF- or non-diabetic ZL-mitochondria, infarct size was significantly decreased (*P* < 0.0001 per each) to 7.0 ± 1.1 and 5.3 ± 1.1% LV area-at-risk, respectively. There was no significant difference in infarct size observed between hearts receiving either diabetic ZDF- or non-diabetic ZL-mitochondria (*P* = 0.999, Fig. 4E,F).

Myocardial infarct size following 30 min. regional ischemia and at 28 days recovery was 36.4 ± 1.1% LV area-at-risk in Vehicle hearts and was significantly decreased to 7.8 ± 1.0% in hearts receiving diabetic ZDF-mitochondria and 4.2 ± 0.5% in hearts receiving non-diabetic ZL-mitochondria (% LV area-at-risk respectively, *P* < 0.0001 per each). There was no significant difference in infarct size observed between hearts receiving either diabetic ZDF- or non-diabetic ZL-mitochondria (*P* = 0.999, Fig. 4E,F).

No peri-infarct expansion was observed within or between hearts receiving either diabetic ZDF- or non-diabetic ZL-mitochondria when comparing 2 h and 28 days recovery (*P* = 0.942).

### Both diabetic ZDF- and non-diabetic ZL-mitochondrial transplantation mitigate myocardial tissue injury

**Figure 5:**
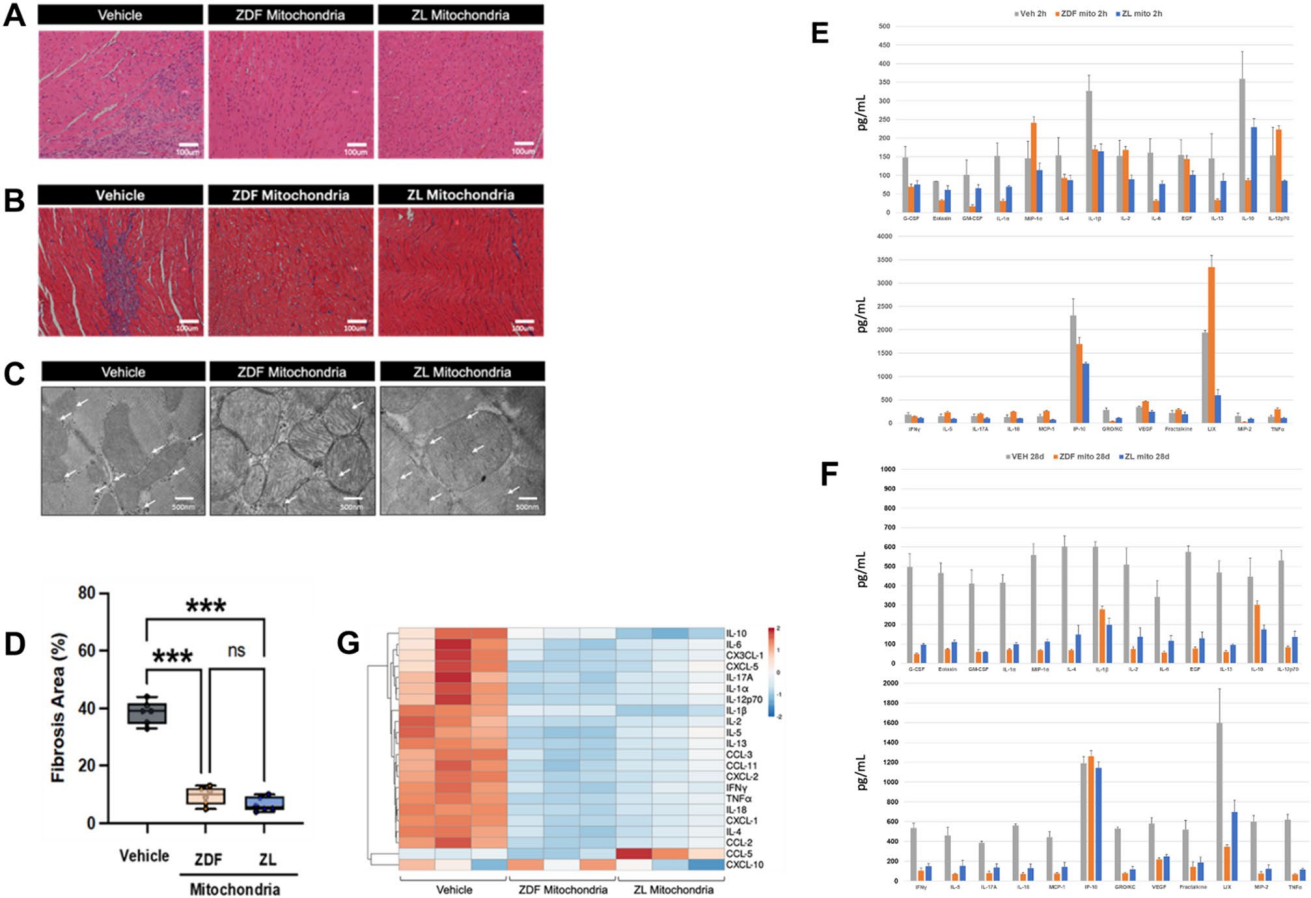
Histology and multiplex analysis. Micrographs of ZDF rat hearts following 30 min. regional ischemia and 2 h (Hematoxylin and Eosin stained) (**A**) and 28 days (Masson’s tri-chrome stained) (**B**) recovery. Hearts receiving vehicle alone (Vehicle) show increased longitudinal and transverse interfibrillar separation as compared to hearts receiving diabetic ZDF -mitochondria and non-diabetic ZL -mitochondria. Representative transmission electron microscopy images (**C**) of ZDF rat hearts 28 days following 30 min of regional myocardial ischemia. Vehicle heart have swollen and electron-translucent mitochondria, with an enlarged intermembrane space, a disrupted matrix and calcium accumulation. Hearts that received either diabetic ZDF- or non-diabetic ZL-mitochondria show only traces of calcium accumulation in mitochondria and preserved mitochondrial structure. White arrows indicate calcium deposits. Magnification 16,000 x. All results are shown as the mean plus and minus the standard error of the mean for n = 6 for each group. Statistical differences are shown. Fibrosis area (**D**) calculated as the percentage of fibrotic tissue area (stained in blue) over the whole area of the myocardium per Masson’s trichrome slide. as *p < 0.05, **p < 0.01, ***p < 0.001; ns: not significant. Multiplex (42-plex) analysis of cytokines and chemokines using the Human Cytokine 42-plex Discovery Assay was performed for hearts following 30 min regional ischemia and 2 h (**E**) and 28 days (**F**) recovery for hearts receiving vehicle alone (Vehicle) or diabetic ZDF- or non-diabetic ZL -mitochondrial transplantation. Significantly increased cytokines as compared to vehicle. **p < 0.001. **G**: Heatmap showing peripheral blood cytokine analysis at 28 days recovery. The log fold change is shown with pseudocolor scale (−2 to 2) with blue denoting down-regulation and orange denoting up-regulation. Samples run in triplicates.

Histological analysis of myocardial tissue within the area-at-risk at 2 h and 28 days recovery showed increased longitudinal and transverse interfibrillar separation in the Vehicle hearts that was not present in hearts receiving diabetic ZDF- or non-diabetic ZL-mitochondria (Fig. 5A–C). Myocardial fibrosis and scar tissue were evident in myocardial tissue within the area-at-risk at 28 days recovery in Vehicle hearts and were significantly decreased in myocardial tissue within the are-at-risk in hearts receiving diabetic ZDF- or non-diabetic ZL-mitochondria at 28 days recovery. Fibrosis area within the area-at-risk was 38.5 ± 1.6% in Vehicle hearts and 9.5 ± 1.3% and 6.5 ± 0.9% in hearts receiving diabetic ZDF- and non-diabetic ZL-mitochondria, respectively (*P* < 0.0001 vs Vehicle per each) (Fig. 5D).

### Mitochondrial transplantation is not associated with any immune or inflammatory response

Multiplex analysis showed that IL-1α, IL-1β, IL-4, IL-6, IL-13, CXCL-1, CXCL-2, CXCL-10, CX3CL-1, TNF-a, and IFNG-γ were significantly decreased in hearts receiving diabetic ZDF- and non-diabetic ZL-mitochondria as compared to Vehicle (*P* < 0.05) suggesting a lack of immunoreactivity to either autologous or heterologous mitochondrial transplantation as well as a mitigation in the myocardial injury (Fig. 5E–G). No late immune or inflammatory response to the transplanted mitochondria was observed by multiplex cytokine analysis in the serum as described above.

### Mitochondrial transplantation upregulates cardiac and muscle contraction pathways

To ascertain the early expressed underlying global transcriptomic changes involved in the cardioprotection conferred by transplantation of diabetic ZDF- or non-diabetic ZL-mitochondria, we performed RNA-sequencing and gene ontology (GO) pathway analysis on tissue isolated from the area-at-risk at 2 h of recovery following 30 min of ischemia.

**Figure 6:**
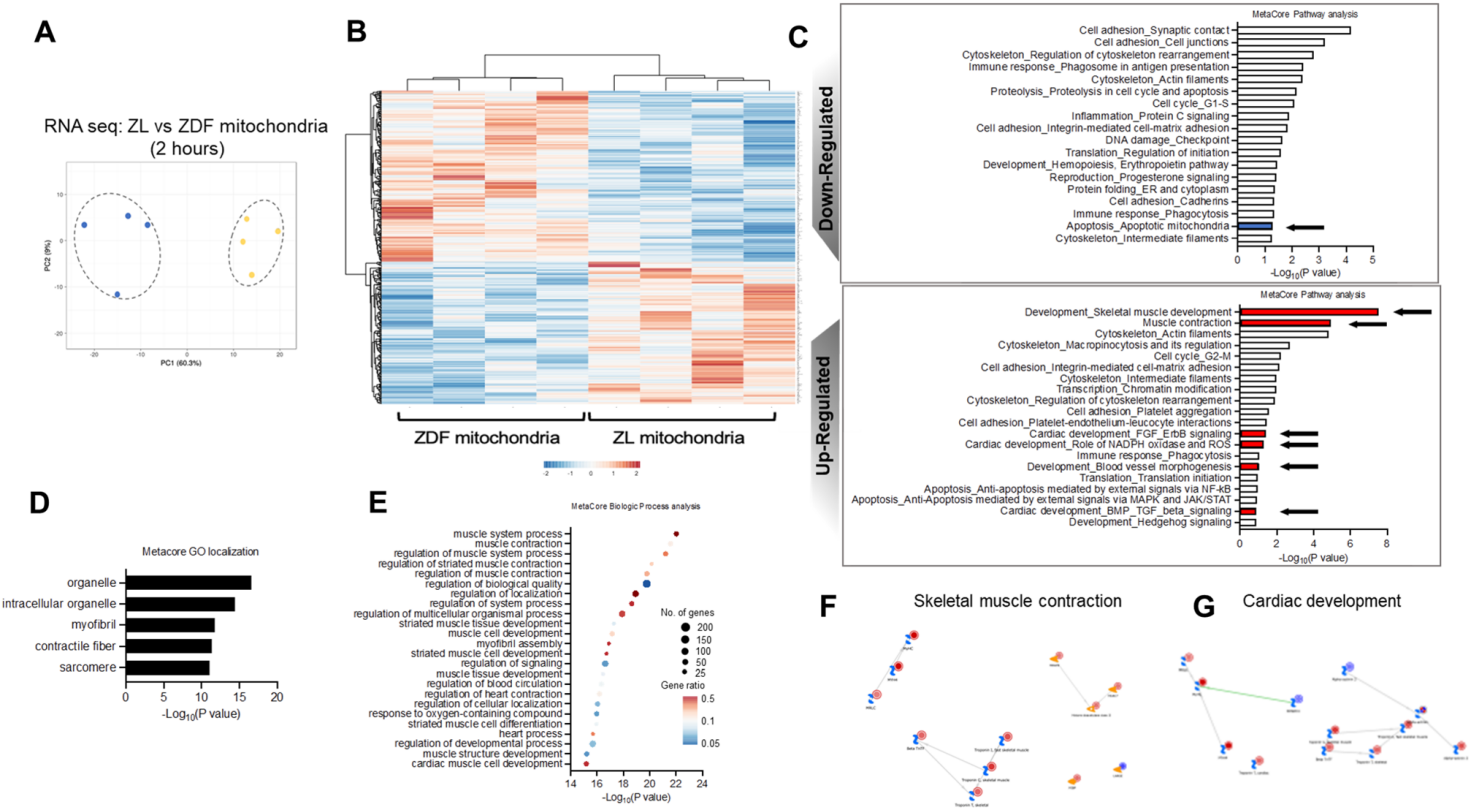
Transcriptomic pathways following 30 min of regional myocardial ischemia and 2 h reperfusion. (**A**) Principal component analysis (PCA) for hearts receiving diabetic ZDF- (gold circles) or non-diabetic ZL -mitochondrial transplantation (blue circles) as compared to hearts receiving vehicle alone. (**B**) Heatmap of transcriptional regulation patterns following 30 min IRI and 2 h recovery, for hearts receiving diabetic ZDF- or non-diabetic ZL -mitochondrial transplantation. Fold changes (FC) are derived from comparison to Vehicle hearts. The log FC of gene expression is shown with pseudocolor scale (− 2 to 2), with blue denoting downregulation and orange denoting upregulation. Columns represent FC comparisons, and rows represent the genes. Experimental groups are found on the bottom. (**C**) A barplot summarizing the top downregulated and upregulated pathways. Colored barplots Red and blue, shown by arrows, indicate the pathways of interest. (**D**) Gene set enrichment analysis using MetaCore localization networks for hearts receiving diabetic ZDF- or non-diabetic ZL -mitochondria. (**E**) Gene set enrichment analysis using MetaCore biologic process analysis of non-diabetic ZL- as compared to diabetic ZDF- mitochondrial transplantation. Color coding indicates the gene ratio (orange higher-blue lower) and size of the dot indicates the number of the genes. Gene network visualization of (**F**) “Skeletal muscle development” and (**G**) “Cardiac development” pathways showing a central node of *troponins*. Node sizes are proportional to the number of dataset proteins present in that pathway node. Node color and scale bars depict the level of betweenness centrality for that pathway node.

Principal component analysis (PCA) indicated that transcriptomic profiles at 2 h recovery, were separate and discrete, for vehicle hearts and hearts receiving non-diabetic ZL- as compared to those receiving diabetic ZDF-mitochondrial transplantation (Fig. 6A).

Comparison of transcripts identified in hearts receiving non-diabetic ZL- as compared to those receiving diabetic ZDF-mitochondrial transplantation at 2 h recovery revealed differentially upregulated pathways in hearts receiving nondiabetic ZL-mitochondrial transplantation. These upregulated pathways include skeletal muscle development and muscle contraction, intermediate filaments cytoskeleton rearrangement, and cardiac development and NADPH (nicotinamide adenine dinucleotide phosphate) and ROS (reactive oxygen species). In addition, nondiabetic ZL-mitochondrial transplantation upregulated signaling pathways for cell cycle regulation, transcription, chromatin modification, anti-apoptosis and macropinocytosis. Downregulated transcriptomic pathways included immune response, proteolysis, inflammation, DNA damage and mitochondrial apoptosis among others (Fig. 6B,C). These transcriptomic alterations are mainly regulated by organelles, intracellular (membrane bound) organelles, myofibrils, contractile fibers, and the sarcomere demonstrating the interaction between the transplanted mitochondria and the muscular excitation system (Fig. 6D).

Analysis of the biological processes affected by nondiabetic ZL-mitochondrial transplantation, were related to muscle biology, namely contraction and development, cytoskeleton regulation, cell adhesion and cardiac development (Fig. 6E).

Bioinformatic analysis indicated that muscle contraction and cardiac development, which highlighted the central role of *troponin*s and *myosin* heavy chain complex, were implicated in the pathophysiological changes of muscle metabolism with nondiabetic ZL-mitochondrial transplantation (Fig. 6F,G).

### Non-diabetic ZL-mitochondrial transplantation transcriptomic alterations are persistent for 28 days following IRI

**Figure 7:**
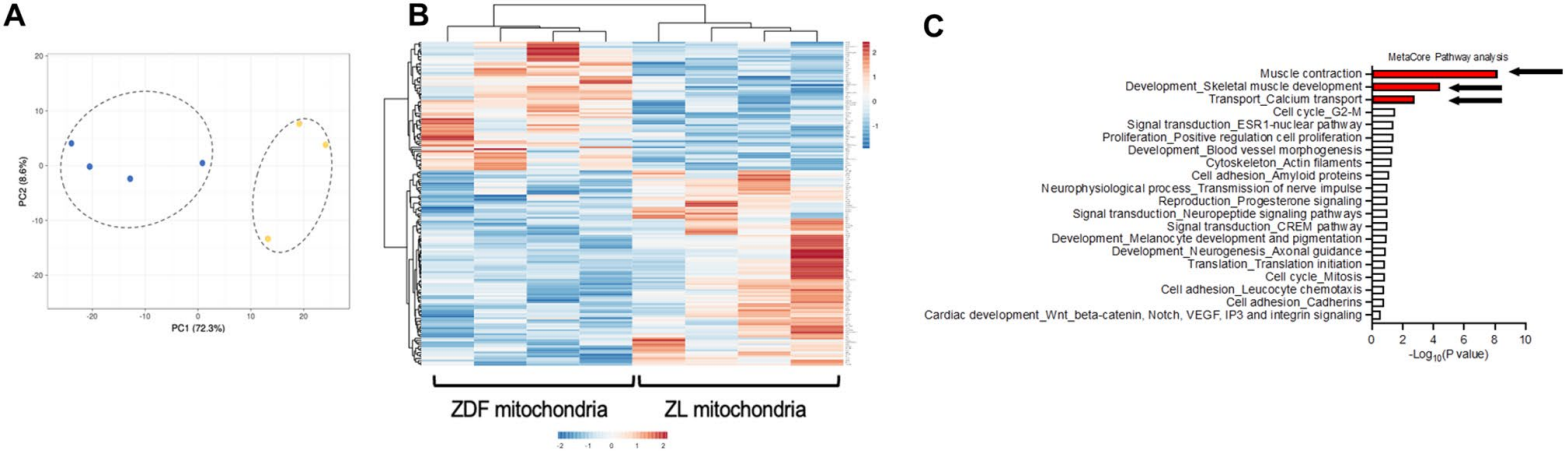
Transcriptomic pathways following 30 min of regional myocardial ischemia and 28 days recovery. (**A**) Principal component analysis (PCA) for hearts receiving diabetic ZDF- (gold circles) or non-diabetic ZL -mitochondrial transplantation (blue circles) as compared to hearts receiving vehicle alone. (**B**) Heatmap of transcriptional regulation patterns following 30 min of IRI and 28 days recovery for hearts receiving diabetic ZDF- (gold circles) or non-diabetic ZL -mitochondrial transplantation (blue circles). Fold changes (FC) are derived from comparison to Vehicle hearts. The log FC of gene expression is shown with pseudocolor scale (− 1 to 2), with blue denoting downregulation and orange denoting upregulation. Columns represent FC comparisons, and rows represent the genes. Experimental groups are found on the bottom. (**C**) Gene set enrichment analysis for hearts receiving diabetic ZDF- or non-diabetic ZL -mitochondrial transplantation. Pathways of interest are shown in red and indicated by arrows.

PCA for RNA-seq samples at 28 days recovery showed discrete transcriptomic profiles between vehicle hearts and hearts receiving non-diabetic ZL- as compared to those receiving diabetic ZDF-mitochondrial transplantation (Fig. 7A).

Comparison of transcripts identified in hearts receiving non-diabetic ZL- as compared to those receiving diabetic ZDF-mitochondrial transplantation at 28 days recovery revealed three differentially upregulated pathways in hearts receiving nondiabetic ZL-mitochondrial transplantation. These upregulated pathways were for muscle contraction and skeletal muscle development and calcium transport (Fig. 7B,C).

**Figure 8:**
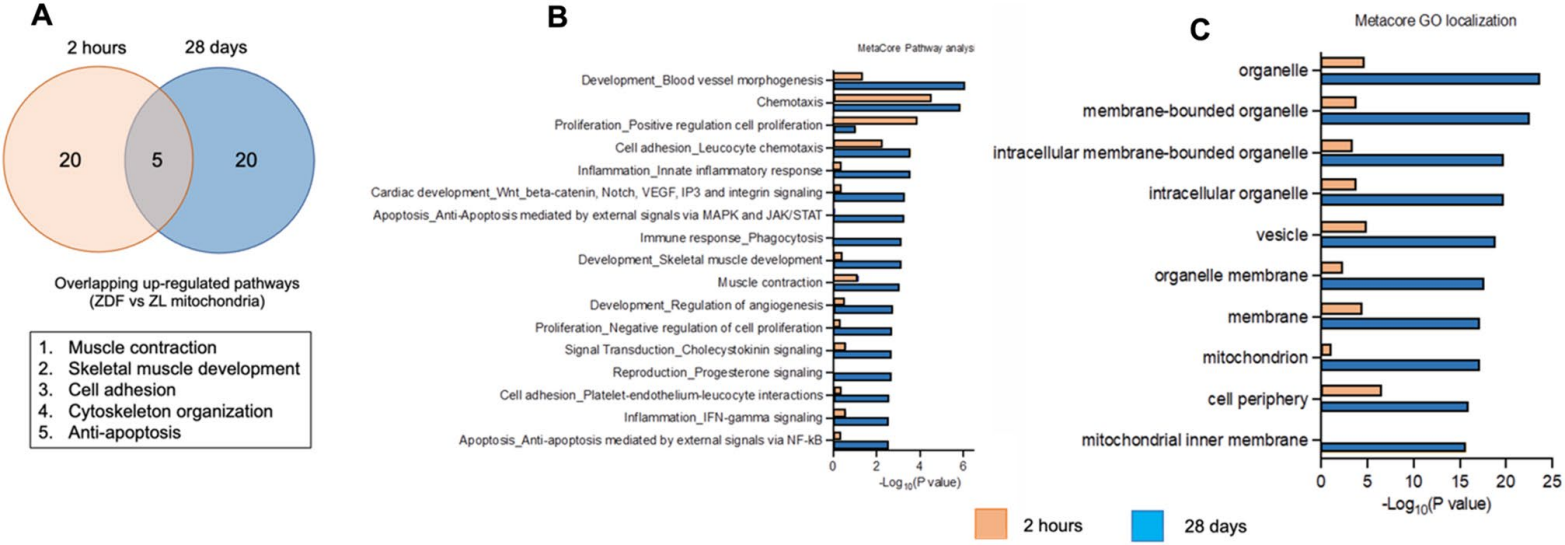
Top 25 upregulated pathways implicated in the transcriptomic differences between hearts receiving diabetic and non-diabetic mitochondrial transplantation. (**A**) Venn diagram of overlapping top 25 MetaCore process networks shows commonality in muscle contraction and development of pathways enriched for hearts receiving diabetic ZDF- and non-diabetic ZL-mitochondrial transplantation compared to Vehicle. (**B**) Gene set enrichment analysis using MetaCore process networks of ZL vs ZDF mitochondria groups combined for 2 h and 28 days. (**C**) Gene set enrichment analysis using MetaCore localization analysis of ZL vs ZDF mitochondria groups combined for 2 h and 28 days.

When overlapping the top 25 upregulated pathways implicated in the transcriptomic differences between hearts receiving diabetic ZDF and hearts receiving non-diabetic ZL- mitochondrial transplantation at 2 h and 28 days recovery, muscle contraction, skeletal muscle development cell adhesion, cytoskeleton organization and anti-apoptosis were shown to be commonly enriched pathways (Fig. 8A).

To assess the contribution of non-diabetic ZL- as compared to diabetic ZDF-mitochondrial transplantation in the observed transcriptomic alterations, a combined analysis was performed. Transplantation of non-diabetic ZL-mitochondria had a more robust effect on biological processes involving inflammation, cardiac development, anti-apoptosis, immune response and skeletal muscle development and contraction, development of angiogenesis as compared to diabetic ZDF-mitochondrial when compared to Vehicle (Fig. 8B). GO localization analysis showed that these processes were affected by the regulation of organelles (membrane bounded, intracellular), mitochondria, the mitochondrial inner membrane and mitochondrial membrane (Fig. 8C).

### Proteomic alterations in cardiomyocyte metabolism are more evident at 28 days in hearts that receive non-diabetic ZL- as compared to diabetic ZDF-mitochondria

**Figure 9:**
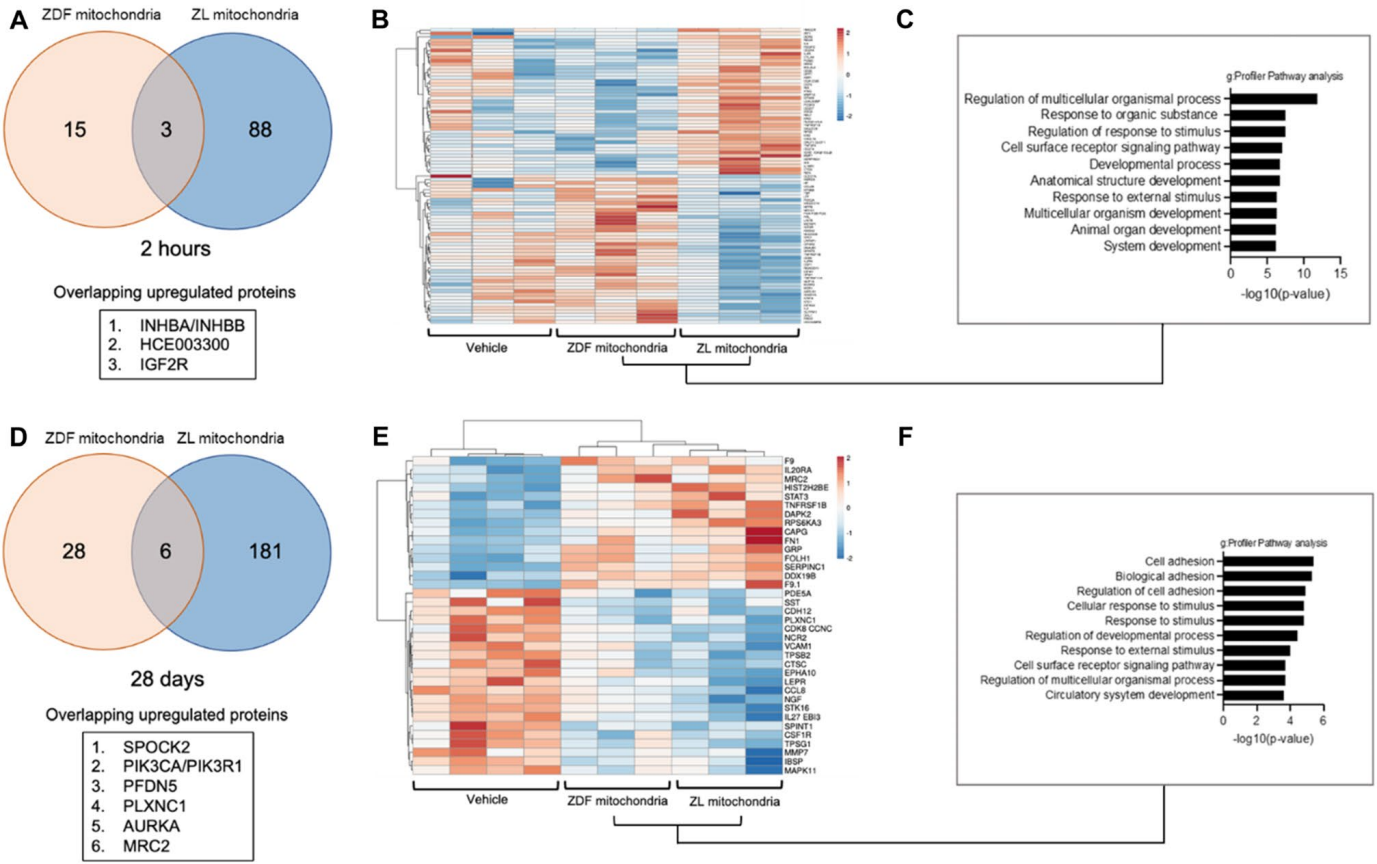
Non-diabetic ZL-mitochondrial transplantation induces a more robust proteomic response compared to ZDF mitochondria both at 2 h and 28 days. (**A**) Venn diagrams of upregulated proteins following 30 min IRI and 2 h recovery as compared to hearts receiving vehicle alone. Fold change is shown > 1.5; p < 0.05. (**B**) Heatmap of protein changes following 30 min IRI and 2 h recovery for hearts receiving diabetic ZDF- or non-diabetic ZL -mitochondrial transplantation as compared to hearts receiving vehicle alone. The log FC of protein levels is shown with pseudocolor scale (− 2 to 2), with blue denoting downregulation and orange denoting upregulation. Columns represent FC comparisons, and rows represent the proteins. Experimental groups are found on the bottom. (**C**) Top 10 enriched process networks following 30 min of IRI and 2 h recovery for hearts receiving diabetic ZDF- or non-diabetic ZL -mitochondrial transplantation as compared to hearts receiving vehicle alone. (**D**) Venn diagrams of upregulated proteins for hearts receiving diabetic ZDF- or non-diabetic ZL -mitochondrial transplantation as compared to hearts receiving vehicle alone following 30 min IRI and 28 days recovery (Fold change > 1.5; p < 0.05). (**E**) Heatmap of protein changes following 30 min IRI and 28 days recovery for hearts receiving diabetic ZDF- or non-diabetic ZL -mitochondrial transplantation as compared to hearts receiving vehicle alone. The log FC of protein levels is shown with pseudocolor scale (− 2 to 2), with blue denoting downregulation and orange denoting downregulation. Columns represent fold change comparisons, and rows represent the proteins. Experimental groups are found on the bottom. Fold change derived from comparison to Vehicle group. (**F**) Top 10 enriched process networks following 30 min IRI and 28 days recovery for hearts receiving diabetic ZDF- or non-diabetic ZL -mitochondrial transplantation as compared to hearts receiving vehicle alone.

To characterize the effect of mitochondrial transplantation on the cardiac tissue, proteomic analysis of the myocardium at risk was performed. Our results showed that following 30 min of RI and 2 h recovery, there were 18 differentially regulated proteins (> 1.5 fold; p < 0.05) in hearts receiving diabetic ZDF-mitochondria and 91 differentially regulated proteins (> 1.5 fold; *P* < 0.05) in hearts receiving non-diabetic ZL-mitochondrial transplantation, as compared to vehicle hearts, with 3 common proteins being differentially expressed (INHBA/NHBB, inhibin subunit beta A; HCE003300, uncharacterized protein; IGF2R, insulin-like growth factor 2 receptor) (Fig. 9A). The proteomic alterations mediated by non-diabetic ZL-mitochondria were distinct at 2 h as demonstrated in the heatmap analysis (Fig. 9B). GO analysis of the biological processes involved in non-diabetic ZL-mitochondrial transplantation included upregulation of multicellular organismal processes, response to organic substance, stimulus and external stimulus, and multicellular organ and system development (Fig. 9C).

At 28 days, there were 34 differentially regulated proteins (> 1.5 fold; *P* < 0.05) in hearts receiving diabetic ZDF-mitochondria and 187 differentially regulated proteins (> 1.5 fold; *P* < 0.05) in hearts receiving non-diabetic ZL-mitochondria, as compared to vehicle hearts, with 6 common proteins being differentially expressed (SPOCK2, cwcv and kazal like domains proteoglycan 2; PIK3CA/PIK3R1, phosphatidylinositol 4,5-bisphosphate 3-kinase catalytic subunit alpha isoform; PFDN5, prefoldin subunit 5; PLXNC1, plexin-C1, AURKA, aurora kinase A; MRC2, C-type mannose receptor 2) (Fig. 9D).

GO analysis showed that non-diabetic ZL-mitochondria transplantation was mediated by upregulation of pathways for cell adhesion, response to stimulus, cell surface receptor signaling, multicellular organismal processes and circulatory system development (Fig. 9E,F).

**Figure 10:**
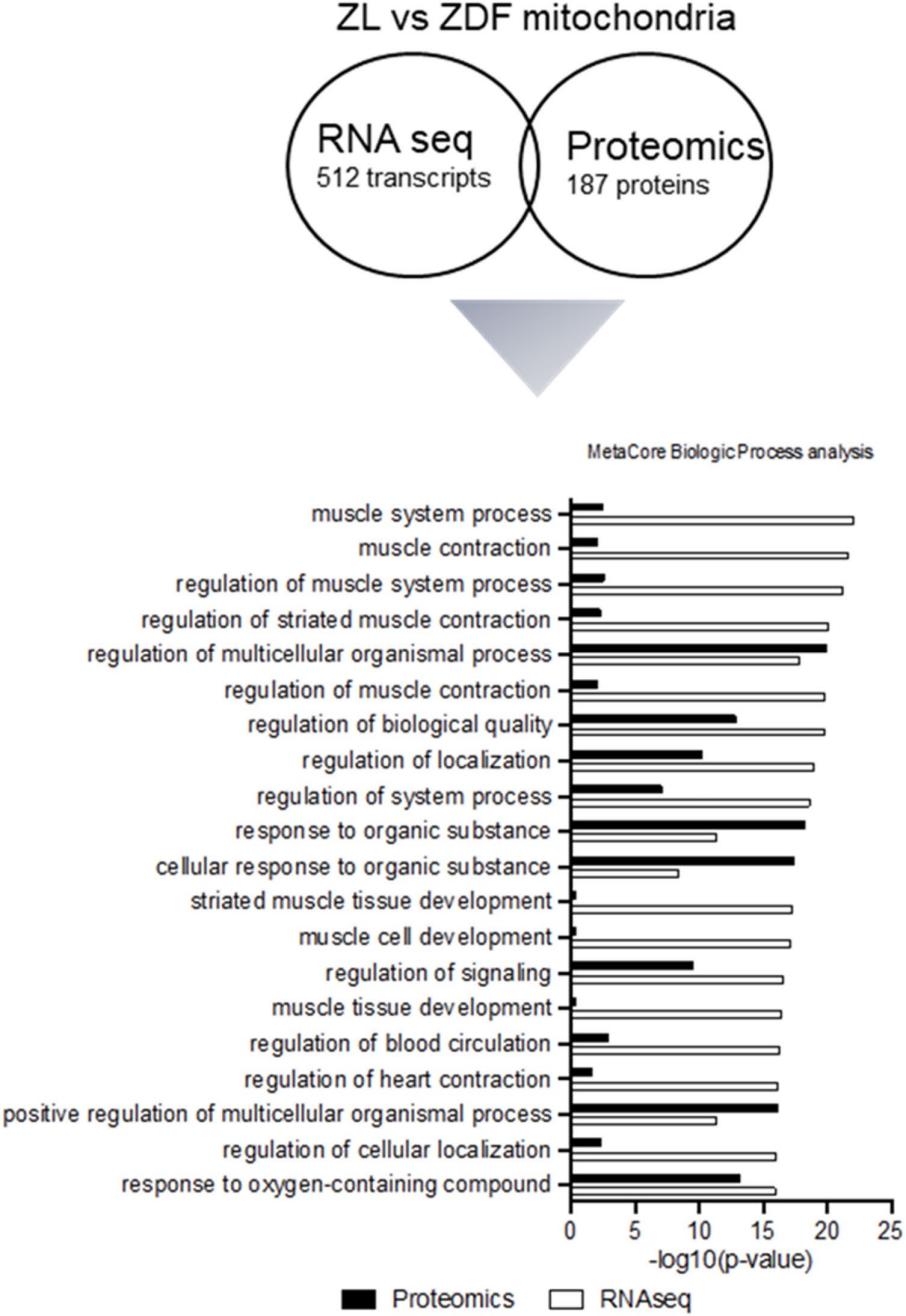
Non-diabetic mitochondrial transplantation transcriptomic changes are consistent with proteomic alterations. Gene ontology-based comparison of RNA-seq (black) and proteomic data (white) using MetaCore in hearts receiving diabetic ZDF- or non-diabetic ZL-mitochondrial transplantation as compared to hearts receiving vehicle alone.

To define whether these transcriptomic changes were consistent with proteomic alterations, combined GO analysis was performed (Fig. 10). Our analysis revealed that biological processes for regulation of multicellular organismal processes, regulation of biological quality, regulation of system processes, regulation of signaling, response to organic substance and response to oxygen containing compound were significantly upregulated in both RNA-seq and proteomic analysis, in hearts receiving non-diabetic ZL-mitochondria, as compared to hearts receiving diabetic ZDF-mitochondria (Fig. 10).

## Discussion

In this investigation we have used the pedigreed Zucker diabetic rat model to investigate the cardioprotective effects of mitochondrial transplantation in the setting of type 2 diabetes^15,16^. The diabetic phenotype of type 2 diabetes was maintained throughout the experimental period. All animals had elevated whole blood glucose and elevated body weight at 28 days recovery. Thus, the observed data and cardioprotective effects are glucose and body weight independent^16,17^.

Our results show that both diabetic and non-diabetic mitochondria significantly decreased post-ischemic myocardial cellular injury and significantly enhanced post-ischemic myocardial function. These results reiterate our previous findings demonstrating the cardioprotective effects of mitochondrial transplantation^2,6–10,12–14^. However, it is important to note that we show that the extent of these cardioprotective effects on post-ischemic myocardial function was attenuated with the use of diabetic ZDF-mitochondria as compared to non-diabetic ZL mitochondria.

We observed no early or late differences in infarct size with either diabetic ZDF- or non-diabetic ZL-mitochondrial transplantation. Both mitochondria populations provided equivalent and lasting enhancement of post-ischemic cellular viability that was evident for at least 28 days recovery with no detectable peri-infarct expansion. These findings were substantiated by histological analysis showing that fibrosis and scar tissue within the area-at-risk was significantly and equally decreased in hearts treated with either diabetic ZDF- or non-diabetic ZL-mitochondria as compared to vehicle hearts.

However, we did observe differences in enhanced post-ischemic myocardial function when we compared diabetic ZDF-mitochondrial transplantation and non-diabetic ZL-mitochondrial transplantation. Both diabetic ZDF- and non-diabetic ZL-mitochondria enhanced post-ischemic myocardial function as compared to hearts receiving vehicle alone, and at one day recovery no difference was observed between hearts receiving either diabetic ZDF- or non-diabetic ZL-mitochondrial transplantation. However, at 1 week recovery we observed a significant attenuation in post-ischemic myocardial function in hearts receiving diabetic ZDF- mitochondria. This attenuation was not observed in hearts receiving non-diabetic mitochondrial transplantation.

The differential efficacy on post-ischemic myocardial function afforded by non-diabetic ZL-mitochondria as compared to diabetic ZDF-mitochondria is likely due to the reduced mitochondrial function of diabetic mitochondria^1–5^. We and others have shown that mitochondrial transplantation improves bioenergetics and oxygen consumption in the myocardium^2,6–10,12–14,18,19^. These beneficial effects are dependent upon the respiratory capacity and integrity of the transplanted mitochondria^6,7,11^. We have shown that in order for cardioprotection to be achieved, the transplanted mitochondria must be intact and respiration competent. The impairment of mitochondrial respiration competency in ZDF-mitochondria is evident in the reduction in total tissue ATP content observed in the area-at-risk. Our results show that total tissue ATP content in the area-at-risk was significantly enhanced with mitochondrial transplantation, however, the absolute increase was significantly decreased in hearts receiving diabetic ZDF-mitochondria as compared to hearts receiving non-diabetic ZL-mitochondria. These findings are in agreement with previous studies suggesting that mitochondrial dysfunction plays a key role in the increased susceptibility of the diabetic myocardium to ischemia- reperfusion injury^2–5,17^. Our results suggest that that mitochondrial dysfunction also plays a role in transcriptomic and proteomic signaling.

RNAseq analysis at 2 h reperfusion showed downregulation of apoptosis and proteolysis and upregulation of anti-apoptosis pathways mediated by external signals via NF-kβ, MAPK and JAK/STAT. NF-kβ, MAPK and JAK/STAT have been shown to activate pathways leading to the activation of anti-apoptotic genes. In previous studies we have shown that the STAT signaling pathway plays an important role in the modulation of apoptosis after ischemia and reperfusion^20^. These studies demonstrated that preservation of mitochondrial structure and function significantly increased anti-apoptotic STAT 3 phosphorylation and its DNA binding and decreased myocardial apoptosis^20^.

The observation that both non-diabetic ZL-mitochondria and diabetic ZDF-mitochondria downregulated immune response and inflammation pathways agrees with our previous findings demonstrating that the transplanted mitochondria do not induce any detectable organ- or systemic immune, autoimmune or inflammatory response. Enzyme-linked immunosorbent spot (ELISpot), enzyme-linked immunosorbent assay (ELISA), fluorescence-activated cell sorting (FACS) and multiplex analysis have failed to detect any immune or inflammatory effects associated with mitochondrial transplantation^6–8,21^. The effect of ROS in this study was not investigated, however, we have previously demonstrated that there is no increase in ROS associated with mitochondrial transplantation^6^.

Multiplex analysis confirmed the above findings and showed that there was a significant decrease in cytokines and chemokines in hearts receiving diabetic ZDF- and non-diabetic ZL-mitochondria as compared to Vehicle hearts. No late immune or inflammatory response to the transplanted mitochondria was observed.

The decrease in transcriptomic and proteomic expression and function as shown by significantly decreased total tissue ATP content by ZDF- as compared to ZL- mitochondria may be associated with enhanced loss of ZDF-mitochondria through mitophagy. We have previously demonstrated that mitochondrial transplantation is not associated with mitophagy. Whether mitophagy occurs with damaged mitochondrial transplantation remains to be elucidated and further studies, and was beyond the scope of this paper.

The absolute levels of transcriptomic and proteomic pathway expression are downregulated with ZDF- as compared to ZL-mitochondrial transplantation and these reduced levels are apparent at 7 days with significantly decreased fractional shortening in ZDF- mitochondria versus ZL-mitochondria hearts. Fractional shortening remained consistent throughout the recovery phase in both ZDF- and ZL-mitochondrial groups. ZDF-mitochondria did not allow for improved fractional shortening with extended reperfusion time suggesting the impaired transcriptomic and proteomic signaling evident at 7 days was not resolved by 28 days reperfusion. This was also in agreement with the reduced ATP content in the myocardial tissue that received ZDF- compared to ZL-mitochondria.

RNAseq analysis at 2 h and at 28 days also showed that in both non-diabetic ZL-mitochondria and diabetic ZDF-mitochondria overlapping upregulated pathways were observed for muscle contraction, skeletal muscle development, cell adhesion, cytoskeletal organization and anti-apoptosis. These overlapping upregulated pathways would allow for early rescue of cell viability shown at 2 h recovery and for the continued preservation of cellular viability observed at 28 days recovery.

SomaScan analysis was in agreement with RNAseq analysis and showed that the number of upregulated proteins was significantly increased in hearts receiving non-diabetic ZL-mitochondria as compared to hearts receiving diabetic ZDF-mitochondria and that this elevated proteomic profile is evident both at 2 h recovery (18 vs. 91, respectively) and at 28 days recovery (34 vs. 187, respectively). These differences are likely due to the decreased respiration competency of the diabetic ZDF-mitochondria and agree with the results of Gomes et al*.*^21^ who demonstrated that downregulation of mitochondrial functional proteins in the diabetic heart with reduced levels of complex I protein subfractions that directly affect mitochondrial electron transport function. These findings are also supported by Mootha et al*.*^22^ who have previously demonstrated that there is down regulation of mitochondria functionally related genes involved in oxidative phosphorylation in human diabetic muscle tissue.

It is important to note that bioinformatic analysis showed the pathways of muscle contraction and cardiac development upregulated by nondiabetic ZL-mitochondrial transplantation and revealed that *troponins* and *myosin* heavy chain and light complexes had a central role. The *troponins* have been shown to play a major role in the modulation of calcium sensitivity and binding in cardiac muscle tissue and would directly affect myocardial contractile efficiency^23^. This would be further enhanced through the upregulation of myosin heavy chain which would enhance actin binding and muscle contraction^23^. Enhanced myosin light chain synthesis or constitution would also allow for enhanced contractile force^24^.

The association of muscle integrin binding protein (MIBP) and histone deacetylase class II would strengthen these assumptions. MIBP functions in the control of myogenic differentiation by regulating integrin-mediated cell interaction and cell adhesion to the extracellular matrix and thus calcium regulation to the myocardium^25^. Upregulation of histone deacetylase class II modulate the expression of genes required for oxygen and glucose utilization and myoblast differentiation^26–28^. a-actinin (ACTN2) is a major structural component of the Z line and plays an important role in maintaining sarcomere integrity and is associated with the structural and functional maturation of cardiomyocytes^29^. Overall, the upregulation of these proteins would allow for enhanced cell rescue and functional recovery of the post-ischemic myocardium.

These studies are not without limitations as they were performed in a rodent model of type 2 diabetes and therefore mimic but do not include all the characteristics and pathways associated with human type 2 diabetes^30,31^. Our ischemia–reperfusion model used a temporary ligation of the LAD with 30 min of warm ischemia time. This time was adopted to ensure animal survival in our vehicle alone group and therefore longer ischemic times would be required for validation in patients where ischemia may be prolonged. The number of mitochondria used in these experiments is based on previous studies showing 2 × 10^5^ – 2 × 10^6^ mitochondria per gram wet weight is required for cardioprotection^6,7,9^. In this study we have used the same number of mitochondria for both diabetic and non-diabetic mitochondrial transplantation to maintain experimental control. Increased mitochondrial number may be required for diabetic mitochondria to reach the observed transcriptomic and proteomic signaling achieved with non-diabetic mitochondria.

In conclusion, we have used a pedigreed rodent model of type 2 diabetes to investigate the cardioprotection afforded by mitochondrial transplantation following temporarily induced regional ischemia and reperfusion and have compared non-diabetic mitochondria and diabetic mitochondria. We show that the transplanted mitochondria are present and functional at 2 h and at 28 days recovery and that both non-diabetic ZL-mitochondria and diabetic ZDF-mitochondria down-regulated immune response and inflammation pathways. Of significance we show that the use of non-diabetic mitochondria for mitochondrial transplantation provides for a more robust preservation of post-ischemic myocardial functional recovery as compared to that achieved using pathologically compromised diabetic mitochondria. These enhanced effects were achieved through the mitochondria dependent upregulation of pathways for cardiac and muscle metabolism and development.

## Methods

### Animal model

Zucker diabetic fatty rats (ZDFLepr^fa^/Crl; Obese fa/fa, ZDF; 396 ± 24 g.) were used for investigation of in vivo T2D myocardial IRI. Non-diabetic Zucker lean rats (Lean + /?, ZL) were used for donor tissue for non-diabetic mitochondrial isolation. All rats were males, aged to 14 weeks old (n = 74), obtained from Charles River Laboratory (Wilmington, MA, USA). When fed the Purina 5008 diet, ZDF rats develop T2D at the age of 12 weeks, which is fully manifested at 14 weeks and lasts up to 24 weeks, while ZL rats maintain normal glucose levels and body weight^2^.

### Non-fasting blood glucose

Non-fasting blood glucose levels were measured by venous sampling from the rat tail vein and tested using the Accu-Chek Mobile blood glucose meter (Roche, Switzerland).

### Experimental model

All animals were housed in groups of three and were subsequently single-housed post-operatively until the end of the study. Water and Purina 5008 diet were provided ad libitum throughout the duration of the study.

The experimental protocol is shown in Fig. 1. In brief, ZDF rats were anesthetized in a chamber with Isoflurane (2–4%) then intubated with an appropriately sized IV catheter and were mechanically ventilated. The animals were given Ketamine (45–75 mg/kg) and Acepromazine: (2.5 mg/kg) IP). Anesthesia was maintained with Isoflurane (1–2%). Following systemic heparinization with unfractionated heparin (100 IU/kg IP), a 1-cm left thoracotomy was performed through the fourth intercostal space. The pericardium was opened, and the left anterior descending artery (LAD) was located and a Prolene thread (6–0) (ETHICON, Somerville, NJ) was passed around the artery with a taper needle, and both ends of the Prolene tie were threaded through a small vinyl tube to form a snare. The LAD was occluded by pulling the snare, which was then fixed by clamping the tube with a mosquito clamp. Regional ischemia in the area-at-risk (AAR) was confirmed, visually by regional cyanosis of the myocardial surface. Regional ischemia was induced for 30 min under anesthesia.

### Mitochondrial Isolation

Donor diabetic ZDF and donor non-diabetic ZL rats were used to obtain pectoralis major muscle for mitochondrial isolation. The donor rats were anesthetized, mechanically ventilated, and heparinized as described above. A 1-cm left thoracotomy was performed through the fourth intercostal space and the pectoralis major located. Two small samples from the pectoralis major were obtained using a 6-mm biopsy punch. Mitochondria were isolated by differential filtration as previously described^3^. The isolated mitochondria were suspended in vehicle solution (250 mmol/l sucrose, 20 mmol/l K + -HEPES (4-(2-hydroxyethyl)-1-piperazine ethane sulfonic acid, pH 7.2), 0.5 mmol/l K + -EGTA (Ethylenediaminetetraacetic acid calcium disodium salt, pH 8.0) and were immediately used for mitochondrial transplantation. Mitochondrial number and viability were determined as previously described^32^.

### Mitochondrial transplantation

Following 30 min of RI, the snare was released, and ZDF rats were randomly assigned to receive either 0.3 mL of vehicle solution alone (Vehicle) or 0.3 mL of vehicle solution containing mitochondria (1 × 10^6^) isolated from either diabetic ZDF rat pectoralis major (ZDF-mitochondria) or mitochondria (1 × 10^6^) isolated from non-diabetic Zucker lean rats (ZL-mitochondria).

Vehicle solution (Vehicle) or the vehicle solution containing mitochondria (diabetic, ZDF- or non-diabetic, ZL-mitochondria) was delivered by epicardial injection directly to the anterior left ventricular wall, in 50 ul injections, into six pre-selected sites in the area at risk using a 1 mL tuberculin syringe with a 32-gage needle. Animals were allowed to recover for 2 h or 28 days.

In the group where animals were allowed to recover for 28 days, the Prolene suture was trimmed and loosely tied and left in place around the LAD to allow for later identification of the area-at-risk. The chest wall and skin incision were closed in layers using a 5–0 PDS* Plus (Antibacterial Monofilament Polydioxanone) and pleural air was evacuated over a needle thoracentesis. The incision was infiltrated with bupivicaine (0.25%, 2–3 mg/kg diluted with saline to 0.125%). Buprenorphine SR (1.2 mg/kg SC, once postoperatively) and Baytril (5 mg/kg SC preoperatively and in 0.2 mg/mL in water for 5–10 days) were delivered after the procedure.

### Transthoracic echocardiography

Transthoracic echocardiography (Vevo 3100, FUJIFILM VisualSonics, Canada) was performed one day before the operation (day 0) and 1, 7, 14, 21 and 28 days post-operatively. During image acquisition, animals were under light anesthesia (3% isoflurane through a nose cone) and body temperature was maintained at 37 ± 0.5° C. M-mode long-axis images were used for left ventricular (LV) end-diastolic (LVEDD) and end-systolic (LVESD) diameter measurements. Fractional shortening (FS) was calculated as the difference between LVEDD and LVESD normalized to LVEDD using a DICOM viewer (Horos Project; https://horosproject.org).

### Mitochondrial uptake

In a separate set of ZDF rats (n = 8), 1 × 10^6^ mitochondria isolated from human cardiac fibroblasts were delivered to the area-at-risk as described above and the rats were allowed to recover for 2 h, 3 days and 28 days. The use of human mitochondria in rodent heart tissue allows for differentiation between endogenous rat mitochondria and transplanted human mitochondria based on immune reactivity. Human mitochondria were detected using a monoclonal anti-human mitochondrial antibody (biotin, MTCO2; Abcam, Cambridge, MA, USA) and visualized using Vectastain (Vector Laboratories, Burlingame, CA, USA) as previously described^7^.

### Tissue harvest, histopathological analysis, and transmission electron microscopy

Following 28 days recovery the animals were re-anesthetized with Ketamine (45–75 mg/kg, IP) and Acepromazine: (2.5 mg/kg, IP). One mL of blood was collected by cardiac puncture for biological analysis and then the chest was opened with an inverted T-shaped incision. The prolene suture around the LAD was secured and the area at risk was delineated by monastryl blue pigment injection into the aorta. The heart was removed, and the animals were euthanized by exsanguination.

Myocardial tissue samples were obtained from the area at risk and fixed in 10% formalin and paraffin-embedded for histopathological analysis. Serial slides were used for hematoxylin and eosin staining and Masson’s trichrome staining as previously described^4^. Fibrosis area was calculated as the percentage of collagen over total area of Masson’s trichrome slides using ImageJ (https://imagej.nih.gov/ij/). Transmission electron microscopy was used to analyze structural damage in the mitochondria, as described in previous studies^4^. All analyses were performed by a blinded observer.

### Measurement of infarct size

Infarct size was determined using 1% triphenyl tetrazolium chloride (Sigma-Aldrich, St. Louis, MO, USA) and the percentage of the AAR was calculated using ImageJ (https://imagej.nih.gov/ij/)^4^. Dry weight-wet weight ratios were determined as previously described ^4^.

### Tissue samples for ATP, protein, RNA seq and SOMAscan analysis

At the end of the recovery period (2 h or 28 days), myocardial tissue samples were obtained from the AAR which was defined by securing the prolene suture around the LAD and injecting monastryl blue pigment injection into the aorta in Vehicle (n = 6), ZDF mitochondria (n = 4), and ZL mitochondria (n = 4) and were snap frozen and then stored in liquid nitrogen prior to ATP, protein, RNA Seq and SOMAscan analysis.

### Total tissue ATP content

Total tissue ATP (adenosine 5′-triphosphate) content in the AAR was determined using the Colorimetric ATP Assay Kit (Abcam) according to the manufacturer’s instructions ^4^.

Myocardial protein per mg wet weight in the AAR was determined using the Pierce BCA Protein Assay Kit (Pierce Biotechnology, Rockford, IL, USA) according to the manufacturer’s instructions. All samples were run in triplicate. ATP content was expressed as mmol/mg protein/mg tissue (wet weight). ATP content was measured at the area at risk, defined as the area delineated after dye injection through the coronaries.

### RNA Seq

RNA Seq analysis was performed after ribodepletion and standard library construction using Illumina HiSeq2500 V4 2 × 100 PE (Genewiz, South Plainfield, NJ). All samples were processed using an RNA-seq pipeline implemented in the bcbio-nextgen project^33^. Raw reads were examined for quality issues using FastQC^34^ to ensure library generation and sequencing were suitable for further analysis. Trimmed reads were aligned to UCSC build mm10 of the Mouse genome, augmented with transcript information from Ensembl release 79 using STAR84. Alignments were checked for evenness of coverage, rRNA content, genomic context of alignments (for example, alignments in known transcripts and introns), complexity, and other quality checks using a combination of FastQC, Qualimap. Counts of reads aligning to known genes were generated by featureCounts85. Differential expression at the gene level were called with DESeq2. The total gene hit counts and CPM values were calculated for each gene and for downstream differential expression analysis between specified groups was performed using DESeq2 and an adapted DESeq2 algorithm, which excludes overlapping reads, called no-overlapping reads. Genes with adjusted False discovery rate (FDR) < 0.05 and log2-fold change (0.5) were called as differentially expressed genes for each comparison. Mean quality score of all samples was 35.67 within a range of 40,000,000–50,000,000 reads per sample. All samples had at least > 70% of mapped fragments over total. MetaCore (v20.2) was used for functional enrichment analysis. MetaCore calculated *P* values for the networks generated based on hypergeometric distribution and evaluated its relevance to gene ontology biological processes.

### SOMAscan

Protein was extracted using T-Per tissue protein extraction agent (Thermo Scientific, USA) per manufacturer’s instructions. Total protein for sample normalization was determined using the Micro BCA Protein Assay Kit (Thermo Scientific, USA). Proteomic profiling was performed using the SomaScan single-stranded DNA aptamer-based platform using SOMAmer reagents^35,36^. Gene ontology enrichment was identified using g:Profiler Rattus Norvegicus database (https://biit.cs.ut.ee/gprofiler) using g:GOSt. Functional enrichment was assessed from ordered list of protein (ascending *p*-value). FDR correction was applied to calculate the adjusted *p*-value.

### Multi-plex assay

Serum samples from day 0 and 2 h and 28 days recovery were used for cytokine and chemokine expression levels using the Bio-PlexT 200 system (Bio-Rad Laboratories, Hercules, Calif) and the Rat Cytokine Array/Chemokine Array 27 Plex (RD27) for Eotaxin, EGF, Fractalkine, IFN-gamma, IL-1 alpha, IL-1 beta, IL-2, IL-4, IL-5, IL-6, IL-10, IL-12(p70), IL-13, IL-17A, IL-18, IP-10, GRO/KC, TNF-alpha, G-CSF, GM-CSF, MCP-1, Leptin, LIX, MIP-1 alpha, MIP-2, RANTES, VEGF. All assays were performed at Eve Technologies (Calgary, Alberta, Canada). All samples were run in triplicates, with standard curves run in duplicates^37^.

### Statistical analysis

The normality of all continuous variables was tested using the Shapiro–Wilk test and graphically assessed by histograms and Q–Q plots. Box-and-whisker plots with super-imposed dot plots were used to illustrate all data except for functional outcome variables (LVESD, LVEDD, FS) that are presented as mean ± standard error of the mean. Between-group comparisons were performed using one-way analysis of variance (ANOVA). Longitudinal analysis for between-groups comparisons was performed using 2-way repeated-measures ANOVA within the framework of fitting mixed-effects linear regression models. To reduce the probability of false-positive results (type I error) due to multiple comparisons, Benjamini and Hochberg’s false discovery rate was applied to control the family-wise error to a < 0.05. All tests reported are 2-tailed. Statistical analyses were performed with GraphPad Prism version 7.0 for Mac OS X (GraphPad Software, La Jolla, CA, USA).

### Ethical approval

All procedures conformed to institutional Guidelines for the Care and Use of Laboratory Animals and were approved by the Institutional Animal Care and Use Committee of Boston Children’s Hospital (Protocol # 18-08-3774) in compliance with the ARRIVE guidelines^38^.

## Data Availability

The datasets used and/or analysed during the current study available from the corresponding author on reasonable request.
